# Trends, relationships and case attribution of antibiotic resistance between children and environmental sources in rural India

**DOI:** 10.1038/s41598-021-01174-w

**Published:** 2021-11-19

**Authors:** Joseph Mitchell, Manju Purohit, Chris P. Jewell, Jonathan M. Read, Gaetano Marrone, Vishal Diwan, Cecilia Stålsby Lundborg

**Affiliations:** 1 Department of Global Public Health, Health Systems and Policy (HSP): Improving Use of Medicines, Karolinska Institutet, 171 77 Stockholm, Sweden; 2 Department of Pathology, R.D. Gardi Medical College, Ujjain, 456006 India; 3 Faculty of Health and Medicine, Lancaster Medical School, Lancaster University, Lancaster, England, UK; 4 Division of Environmental Monitoring and Exposure Assessment (Water and Soil), ICMR – National Institute for Research in Environmental Health, Bhopal, 462030 India

**Keywords:** Microbiology, Health care

## Abstract

Bacterial antibiotic resistance is an important global health threat and the interfaces of antibiotic resistance between humans, animals and the environment are complex. We aimed to determine the associations and overtime trends of antibiotic resistance between humans, animals and water sources from the same area and time and estimate attribution of the other sources to cases of human antibiotic resistance. A total of 125 children (aged 1–3 years old) had stool samples analysed for antibiotic-resistant bacteria at seven time points over two years, with simultaneous collection of samples of animal stools and water sources in a rural Indian community. Newey–West regression models were used to calculate temporal associations, the source with the most statistically significant relationships was household drinking water. This is supported by use of *SourceR* attribution modelling, that estimated the mean attribution of cases of antibiotic resistance in the children from animals, household drinking water and wastewater, at each time point and location, to be 12.6% (95% CI 4.4–20.9%), 12.1% (CI 3.4–20.7%) and 10.3% (CI 3.2–17.3%) respectively. This underlines the importance of the ‘one health’ concept and requires further research. Also, most of the significant trends over time were negative, suggesting a possible generalised improvement locally.

## Introduction

Antibiotic resistance of bacteria threatens the core of modern medicine^1–3^ and is an important global health threat. However, the increasing exposure to antibiotics, due to excessive use of antibiotic agents in agriculture and healthcare, contribute to rising bacterial antibiotic resistance^1,4^. The development of resistance occurs not only in pathogens, but also in commensal bacteria^5^ such as *Escherichia coli (E. coli)*, which act as a reservoir for resistance genes^6^. The use of antibiotics not only applies selective pressure towards antibiotic resistance, but also stimulates transfer of resistance determinants between microorganisms^4^ and the resistance can spread to pathogens^7,8^.

The complex and interlinking drivers of antibiotic resistance have led international organisations and research communities to recommend a ‘one-health’ approach; a collaboration of health science and food and agriculture professions to attain optimal health for humans, animals and the environment^1,9^. Antibiotic misuse, poor antibiotic quality, lack of antibiotic stewardship, poor infection control procedures and a lack of surveillance and governance act as risk factors for antibiotic resistance^7,10^. Risk factors for onward transmission include a lack of sanitation, inadequate clean water access, low standards of infection control, poor access to diagnostics, migration, travel and reduced access to quality antibiotics^4^. Antibiotic resistance can be transmitted from human to human and from the environment or animals to humans and vice versa^11^. The pathways are complex, with the environment acting as an important reservoir of resistance^12^, and direct and indirect contact with animals can transmit resistance^7,12,13^. Antibiotic residues and resistance have also been found in various water sources, both natural and human-made, across the world^14–22^. In countries and communities where water is sourced from groundwater or untreated surface water there is increased risk of exposure to antibiotic resistance and harm to human health^23^. It is suggested that drinking water may contribute to development of resistance in humans^23^ and it is likely to be of more importance in countries with poor sewage and water treatment^9^.

There is considerable variation of antibiotic resistance globally, but it is a problem that concerns every country^24^. It is predicted that most deaths in the future from antibiotic resistance will be in Africa and Asia, with India, Nigeria, Indonesia and Russia particularly at risk^24^. The level of antibiotic resistance in India is increasing^25^, but there is insufficient surveillance of the resistance trends, with particular scarcity in rural settings^25–27^.

Mathematical modelling has already been used to help understand antibiotic resistance transmission networks between the three domains of the ‘one health’ concept^28^. However, there are difficulties in applying mathematical models to processes as complicated and inexact as antibiotic resistance. Models need to incorporate factors across the diverse biospheres^28^ underlining the importance in the ‘one-health’ concept, even when primarily considering human health. *SourceR* is an open-access statistical model that considers all aspects of ‘one health’ and has prior identified chickens as the most important source of a human campylobacteriosis outbreak^29^.

There has been substantial work in improving knowledge of drivers and transmission pathways of antibiotic resistance. However, the exact relationships between resistance in humans and animals^7,30^ and humans and their environment remain unclear^7,23^. Therefore, we aimed to determine the temporal associations and over time trends of antibiotic resistance of commensal *E. coli* isolated from children and their household drinking water, source drinking water, wastewater and from animals sharing the same community environment in rural India, over a 2-year period. This was supported by the application of *SourceR* to assess attribution of resistance.

## Results

### Overall level of antibiotic resistance

With the exception of colistin, *E.coli* isolated from all of the sources exhibited resistance to all antibiotics at one point in the study. There was no identified colistin resistance in animals, source drinking water and wastewater sources. The overall range of prevalence of resistance to individual antibiotics at each time point in humans and the measured sources varied greatly from 0.0 to 97.2%, see Supplementary Table 1. An overview of the pattern of resistance (see Supplementary Fig. 1), shows that overall resistance prevalence were higher in human samples (24.7%) than the other sources (10.0%, 11.7%, 14.6% and 15.5% in animals, household drinking water, source drinking water and wastewater respectively).

### Temporal association of human antibiotic resistance to that of the other sources

For Newey–West regression model calculated associations between human proportions of antibiotic resistance and the other sources (n = 69 analyses done), 18 combinations of antibiotic and source showed a statistically significant association between the two groups (see Table 1). Colistin was excluded from analysis for the animal, source drinking water and wastewater groups as no resistance was seen in any of the three time-points it was measured. In this analysis, the regression coefficients relate to the statistical percentage increase/decrease in the dependent variable (human) as the percentage of independent variable increases/decreases for each time point. Of the 18 statistically significant associations between human and other source, three were with village animals (ampicillin, nalidixic acid and imipenem), eight with household drinking water sources (ampicillin, cefotaxime, cefepime, tetracycline, imipenem, sulfamethoxazole, extended spectrum beta-lactamase (ESBL) and multi-drug resistance (MDR)), two with source drinking water (ampicillin and imipenem) and five with wastewater (ampicillin, cefotaxime, tigecycline, imipenem and co-trimoxazole). Both ampicillin and imipenem had statistically significant associations between human resistance levels and all other sources measured.

**Table 1:** Temporal associations between the proportion of resistance to antibiotics in children aged 1–3 years old and that of animals, household water, source drinking water and wastewater within the same community.

Antibiotics	Animal		Household water		Source drinking water		Wastewater	
	Regression coefficient (95% CI)	*p*-value	Regression coefficient (95% CI)	*p*-value	Regression coefficient (95% CI)	*p*-value	Regression coefficient (95% CI)	*p*-value
Ampicillin	**58.70 (44.96** to **72.43)**	**0.000**	**66.26 (57.88** to **74.63)**	**0.000**	**72.54 (60.14** to **84.93)**	**0.000**	**64.89 (51.23** to **78.54)**	**0.000**
Cefotaxime	6.41 (−126.60 to 139.43)	0.906	**120.33 (101.43 **to **139.23)**	**0.000**	−3.58 (−58.07 to 50.91)	0.872	−**22.25 (**−**39.65 **to −**4.84)**	**0.022**
Ceftazidime	−6.15 (−129.58 to 117.29)	0.903	225.84 (−180.89 to 632.57)	0.213	−6.36 (−65.19 to 52.47)	0.792	−13.05 (−31.69 to 5.59)	0.132
Cefepime	245.82 (−54.20 to 545.83)	0.089	**163.14 (62.10 **to **264.18)**	**0.009**	56.75 (−30.59 to 144.09)	0.156	56.45 (−15.65 to 128.55)	0.100
Nalidixic acid	−**71.50 (**−**133.68 **to −**9.32)**	**0.032**	63.50 (−20.52 to 147.52)	0.110	6.26 (−23.51 to 36.03)	0.612	4.74 (−30.78 to 40.27)	0.745
Ciprofloxacin	−106.28 (−232.01 to 19.44)	0.082	56.94 (−3.33 to 117.20)	0.059	−4.35 (−106.35 to 97.85)	0.919	−11.35 (63.92 to 41.23)	0.603
Nitrofurantoin	15.03 (−36.95 to 67.01)	0.491	31.88 (−17.64 to 81.40)	0.159	−4.63 (−27.56 to 18.30)	0.626	−1.56 (−91.20 to 88.08)	0.966
Gentamicin	19.42 (−85.78 to 124.62)	0.655	11.19 (−58.84 to 81.22)	0.698	2.20 (−14.98 to 19.38)	0.755	34.24 (−32.53 to 101.01)	0.245
Amikacin	48.48 (−38.95 to 135.91)	0.213	29.86 (−45.76 to 105.49)	0.357	2.61 (−11.13 to 16.35)	0.646	5.71 (−117.25 to 128.68)	0.910
Tetracycline	−21.36 (−122.28 to 79.56)	0.610	**93.49 (0.55 **to **186.44)**	**0.049**	4.72 (−15.15 to 24.58)	0.568	41.35 (−18.82 to 101.53)	0.138
Tigecycline	−5.34 (−28.67 to 17.99)	0.582	−17.43 (−50.08 to 15.22)	0.228	0.91 (−2.99 to 4.82)	0.573	**8.40 (0.43 **to **16.36)**	**0.042**
Imipenem	**66.52 (21.81** to **111.22)**	**0.012**	**111.20 (104.51 **to **117.89)**	**0.000**	**60.74 (37.91 **to **83.58)**	**0.001**	**81.03 (32.79 **to **129.27)**	**0.008**
Meropenem	−217.62 (−471.84 to 36.59)	0.079	121.83 (−104.19 to 347.85)	0.224	1.37 (−29.15 to 31.89)	0.913	18.08 (−16.52 to 52.67)	0.237
Co- trimoxazole	−80.23 (−240.44 to 79.98)	0.254	96.96 (−21.64 to 215.57)	0.090	−26.31 (−103.57 to 50.95)	0.421	−**61.37 (**−**107.95 **to −**15.80)**	**0.020**
Sulphamethiazole	−36.31 (−97.60 to 24.97)	0.188	**112.84 (19.15 **to **206.53)**	**0.027**	−19.14 (−57.11 to 18.83)	0.252	−15.48 (−47.64 to 16.67)	0.271
Colistin	N/A (N/A)	N/A	−4.72 (−55.54 to 46.09)	0.447	N/A (N/A)	N/A	N/A (N/A)	N/A
ESBL	−63.78 (−204.33 to 76.78)	0.296	**201.07 (123.72 **to **278.41)**	**0.001**	15.58 (−35.07 to 66.22)	0.465	−10.85 (−47.29 to 25.60)	0.479
MDR	−35.11 (−164.73 to 94.50)	0.517	**118.45 (9.42 **to **227.49)**	**0.038**	−26.92 (−58.58 to 4.75)	0.081	−35.87 (78.26 to 6.53)	0.082

*ESBL* extended spectrum beta lactamase, *MDR* multi drug resistant.

Bold text indicates statistical significance (p-value <0.05).

### Case attribution of human antibiotic resistance from other sources

**Figure 1:**
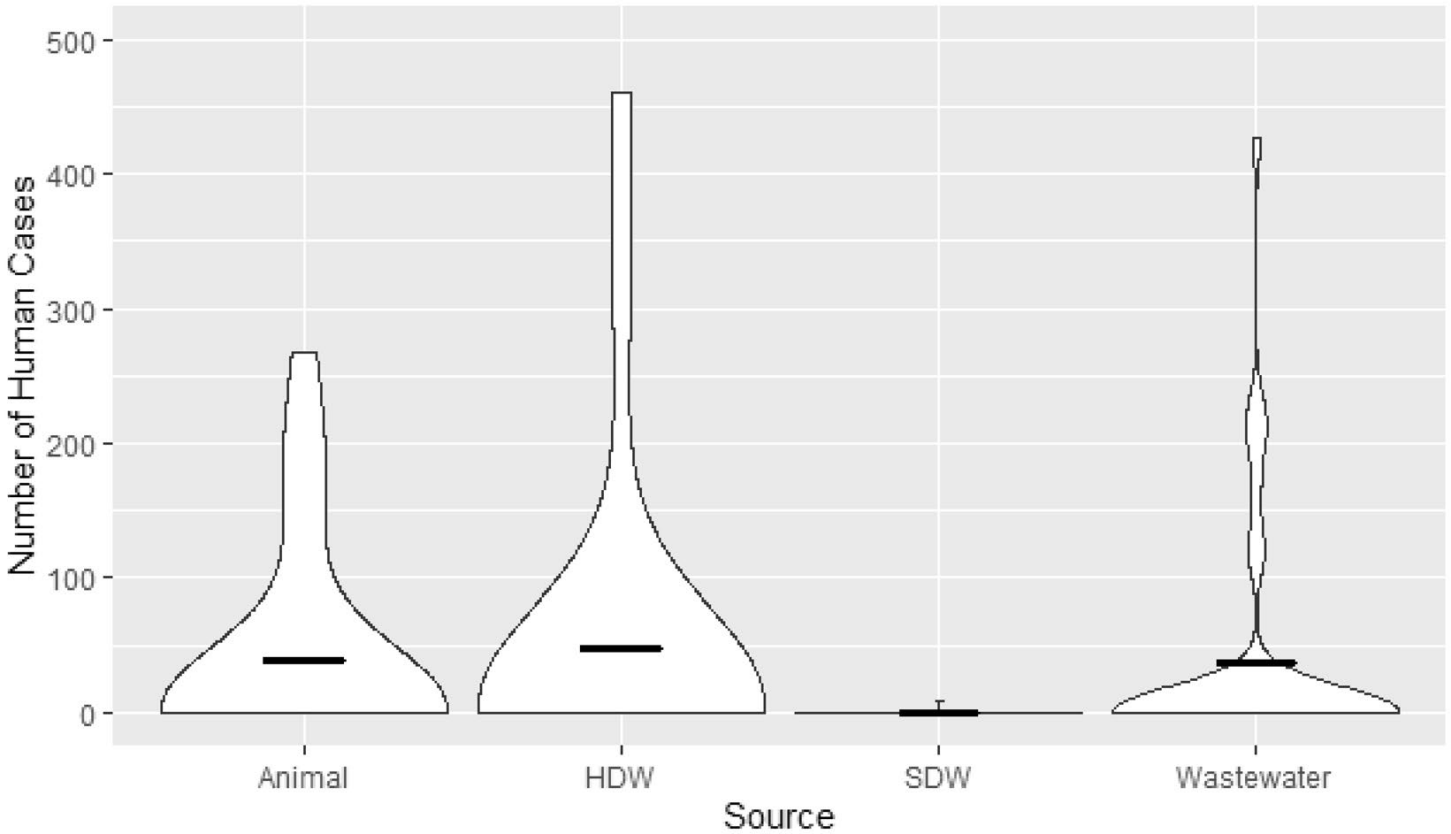
Violin Plot of the proportion of Human Cases Attributable to Each Environmental Source. The bold horizontal line for each source represents the mean for each source. The bold horizontal line for each source represents the mean for each source. HDW Household Drinking Water, SDW Source Drinking Water.

**Figure 2:**
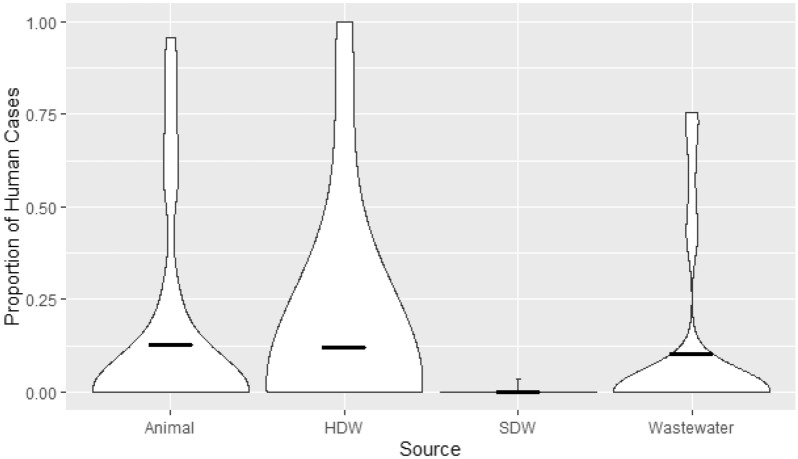
Violin Plot of the proportion of Human Cases Attributable to Each Environmental Source. The bold horizontal line for each source represents the mean for each source. HDW Household Drinking Water, SDW Source Drinking Water.

The attribution modelling with *SourceR* estimated that more human isolates were more associated with those in animals, human drinking water and wastewater than source drinking water. The mean of human cases attributable to each environmental source at each time point and location was 38.3 (95% Confidence Interval (CI) 14.3–62.3), 47.7 (CI 10.8–84.6) and 36.7 (CI 9.0–64.4) cases for animals, household drinking water and wastewater respectively, but it was only 0.2 (CI − 0.2–0.67) for source drinking water (Fig. 1). The mean of the percentage of human cases attributable to each source at each time point and location was found to be 12.6% (CI 4.4–20.9%), 12.1% (CI 3.4–20.7%) and 10.3% (CI 3.2–17.3%) for animals, human drinking water and wastewater respectively; for source drinking water it was 0.1% (CI − 0.1–0.2%) (Fig. 2).

### Temporal trends of antibiotic resistance in humans and other sources

**Figure 3:**
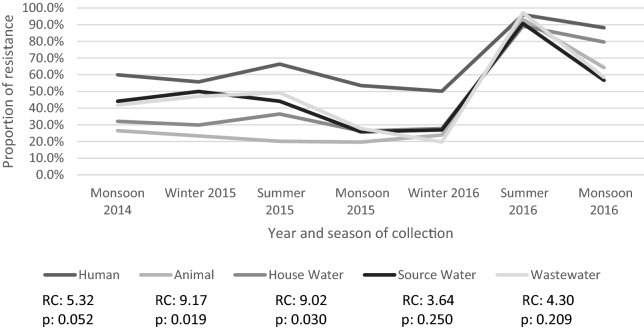
Line graph of over time trend of percentage of resistance to ampicillin, with regression coefficients and *p*-value for Newey–West analyses. *RC* Regression Coefficient *p* = *p*-value.

**Figure 4:**
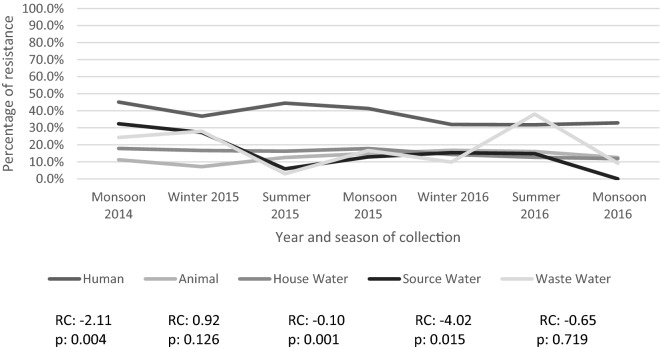
Line graph of over time trend of percentage of production of ESBL, with regression coefficients and *p*-value for Newey–West analyses. *RC* Regression Coefficient *p* = *p*-value.

Of the temporal trends of percentage of antibiotic resistance (n = 87), 23 showed a statistically significant trend (see Table 2). Again, colistin was excluded from analysis for the animal, source drinking water and wastewater groups as no resistance was seen in any of the three time points it was measured. The regression coefficient relates to the trend of increase or decrease in resistance as a percentage per time unit (four months). Of these statistically significant trends, two were in human samples (amikacin and ESBL), five in animal samples (ampicillin, cefepime, gentamicin, amikacin and sulfamethoxazole), four in household water samples (ampicillin, gentamicin, tetracycline and ESBL), seven in source drinking water (cefotaxime, nalidixic acid, ciprofloxacin, tetracycline, meropenem, ESBL and MDR) and five in wastewater samples (nalidixic acid, ciprofloxacin, gentamicin, tigecycline and sulfamethoxazole). Of the 23 statistically significant results, five had an increasing and 18 a decreasing trend. In the antibiotic groups that had more than one statistically significant trend, the direction of the regression coefficient was the same in each source: ampicillin, see Fig. 3, (animal and household drinking water, increase), nalidixic acid (source drinking water and wastewater, decrease), ciprofloxacin (source drinking water and wastewater, decrease), gentamicin (animal, household drinking water and wastewater, decrease), amikacin (human and animal, increase), tetracycline (household drinking water and source drinking water, decrease), sulfamethoxazole (animal and wastewater, decrease) and ESBL, see Fig. 4, (human, household drinking water and source drinking water, decrease). The other statistically significant regression coefficients were cefotaxime (source drinking water, decrease), cefepime (animal, increase), tigecycline (wastewater, decrease) and meropenem (source drinking water, decrease).

**Table 2:** Temporal trends of antibiotic resistance detected in humans, animals, household drinking water, source drinking water and wastewater, for each antibiotic.

Antibiotics	Human		Animal		Household drinking water		Source drinking water		Wastewater	
	Regression coefficient (95% CI)	*p*-value	Regression coefficient (95% CI)	*p*-value	Regression coefficient (95% CI)	*p*-value	Regression coefficient (95% CI)	*p*-value	Regression coefficient (95% CI)	*p*-value
Ampicillin	5.32 (−0.08 to 10.71)	0.052	**9.17 (0.67 **to **17.77)**	**0.039**	**9.02 (1.23 **to **16.75)**	**0.030**	3.64 (−3.56 to 10.84)	0.250	4.30 (−3.37 to 11.97)	0.209
Cefotaxime	−0.57 (−2.86 to 1.71)	0.547	0.17 (−1.48 to 1.82)	0.802	−0.90 (−1.92 to 0.13)	0.075	−**3.61 (**−**6.84** to −**0.39)**	**0.035**	0.33 (−4.78 to 5.43)	0.875
Ceftazidime	0.46 (−2.21 to 3.13)	0.677	0.84 (−1.02 to 2.71)	0.297	−0.02 (−0.33 to 0.29)	0.881	−3.49 (−6.98 to 0.01)	0.050	0.97 (4.11 to 6.06)	0.643
Cefepime	2.32 (−3.13 to 7.77)	0.324	**0.85 (0.11 **to **1.58)**	**0.032**	0.46 (−2.38 to 3.30)	0.696	−1.03 (−7.52 to 5.47)	0.701	2.63 (−2.01 to 7.27)	0.205
Nalidixic acid	−0.46 (−2.49 to 1.58)	0.588	0.61 (−1.46 to 2.67)	0.482	−0.76 (−1.83 to 0.31)	0.129	−**6.02 (**−**7.48 **to −**4.56)**	**0.000**	−**3.71 (**−**5.88 **to −**1.55)**	**0.007**
Ciprofloxacin	0.39 (−2.18 to 2.96)	0.711	−0.52 (−1.86 to 0.81)	0.355	−0.96 (−1.94 to 0.02)	0.054	−**2.39 (**−**2.74 **to −**2.03)**	**0.000**	−**2.19 (**−**4.13 **to −**0.25)**	**0.033**
Nitrofurantoin	−0.38 (−1.03 to 0.27)	0.191	−0.91 (−2.08 to 0.26)	0.103	0.09 (−1.77 to 1.95)	0.906	−0.52 (−2.34 to 1.30)	0.493	0.48 (−0.29 to 1.24)	0.172
Gentamicin	−0.22 (−0.61 to 0.17)	0.206	−**0.20 (**−**0.37 **to −**0.03)**	**0.03**	−**0.52 (**−**0.77 **to −**0.27)**	**0.003**	−0.11 (−1.35 to 1.14)	0.837	−**0.41 (**−**0.79 **to −**0.03)**	**0.040**
Amikacin	**0.43 (0.07 **to **0.79)**	**0.028**	**0.62 (0.26 **to **0.98)**	**0.007**	−0.18 (−0.61 to 0.24)	0.320	−0.95 (−3.07 to 1.18)	0.304	0.28 (−0.17 to 0.72)	0.172
Tetracycline	−0.17 (−1.87 to 1.53)	0.805	−0.57 (−2.05 to 0.92)	0.372	−**1.10 (**−**1.97** to −**0.26)**	**0.023**	−**6.36 (**−**8.44 **to −**4.28)**	**0.001**	−1.37 (−3.44 to 0.69)	0.148
Tigecycline	−0.02 (−0.08 to 0.04)	0.434	0.05 (−0.39 to 0.49)	0.780	−0.07 (−0.20 to 0.05)	0.186	−1.27 (−2.55 to 0.01)	0.051	−**0.35 (**−**0.67 **to −**0.04)**	**0.033**
Imipenem	−0.05 (−5.30 to 5.20)	0.980	−0.84 (−6.32 to 4.64)	0.710	−0.15 (−4.76 to 4.46)	0.937	−1.30 (−7.00 to 4.36)	0.581	−0.46 (−4.89 to 3.98)	0.802
Meropenem	1.22 (−1.74 to 4.19)	0.337	−0.32 (−1.08 to 0.43)	0.319	−0.38 (−1.89 to 1.13)	0.543	−**4.14 (**−**7.52 **to −**0.77)**	**0.025**	−1.35 (−3.65 to 0.96)	0.193
Co-trimoxazole	1.06 (−0.51 to 2.63)	0.144	−0.14 (−1.02 to 0.73)	0.688	0.12 (−0.87 to 1.10)	0.773	−0.77 (−2.04 to 0.49)	0.177	−0.71 (−3.16 to 1.73)	0.487
Sulphamethiazole	0.23 (−1.79 to 2.24)	0.785	−**2.95 (**−**4.45 **to −**1.46)**	**0.004**	−0.74 (−2.27 to 0.78)	0.266	−2.73 (−6.88 to 1.43)	0.153	−**2.52 (**−**4.75 **to −**0.29)**	**0.034**
Colistin	0.07 (−0.16 to 0.29)	0.167	N/A (N/A)	N/A	−0.47 (−4.44 to 3.51)	0.375	N/A (N/A)	N/A	N/A (N/A)	N/A
ESBL	−**2.11 (**−**3.21 **to −**1.01)**	**0.004**	0.92 (−0.37 to 2.20)	0.126	−**0.10 (**−**1.38 **to −**0.61)**	**0.001**	−**4.02 (**−**6.88** to −**1.15)**	**0.015**	−0.65 (−5.05 to 3.75)	0.719
MDR	1.22 (−1.07 to 3.50)	0.229	0.08 (−2.66 to 2.83)	0.940	−0.47 (−1.76 to 0.83)	0.398	−**5.50 (**−**7.36 **to −**3.64)**	**0.001**	−0.70 (−4.56 to 3.16)	0.661

*ESBL* extended spectrum beta lactamase, *MDR* multi drug resistant.

Bold text indicates statistical significance (p-value <0.05).

## Discussion

We conducted an examination of antibiotic resistance in human (young children) stools, village animals’ stools, household drinking water, source drinking water and wastewater in a rural Indian community. We have investigated, for the first time, temporal associations between antibiotic resistance in humans and the other sources. Previous studies, including a meta-analysis, have compared prevalence of resistance of simultaneously collected specimens from humans, animals and the environment. However, they have not explored the association between them, often due to methodological limitations^30–32^. Of the analyses of temporal association between sources (n = 69), 18 were statistically significant and the vast majority of these were positively associated. Of the significant associations, most were with household drinking water, suggesting a stronger relationship between human antibiotic resistance and that of their household drinking water compared to the other sources. This is in accordance with the review of antibiotic resistance in drinking water systems that concluded that the consumption of contaminated water may contribute to antibiotic resistance in humans^23^. However, with regards to the temporal association calculations, our results cannot be used to indicate direction of transmission or direct causality. Nevertheless, given that all sources showed some statistically significant associations to human antibiotic resistance in the Newey–West regression models, further research is needed to greater understand the level and importance of the associations between humans and the animals and environmental sources local to them. Our findings are supported by our previous analysis of the same data, where similar patterns of co-resistance and gene carriage were identified between each of the sources^11^. As this is the first study that quantitatively analyses temporal association, in this case using Newey–West regression modelling, between humans and environmental sources, further research is also required to investigate whether our results are repeatable and transfer to other locations, particularly where humans have different interactions with animals and their environment. A study from Europe found a mirroring of resistance patterns in wastewater samples and clinical samples in the same area, but no measure of association was made^14^. Furthermore, this study was from wastewater plants rather than open wastewater as we studied, which makes direct comparison difficult.

The findings of the calculations of temporal association were supported by the results when we applied the attribution model, *SourceR*. The case attribution results estimated that most human cases of antibiotic resistance were attributed to household drinking water per time point and location (mean = 47.7 (CI 10.8–84.6) cases of antimicrobial resistance in each village at each time point). This was closely followed by animal (38.3, CI 14.3–62.3) and wastewater (36.7, CI 9.0–64.4), however source drinking water attributed to only 0.2 (CI − 0.2–0.67) cases. A similar pattern of attribution was seen when considering the proportion of human cases attributed to each source per time point and location. However, the source with the highest percentage of cases attributed was animals (12.6% (CI 4.4–20.9%)), then household drinking water and wastewater (12.1% (CI 3.4–20.7%)) and 10.3% (CI 3.2–17.3%) respectively). Again, the lowest level of case attribution was seen in source drinking water (0.1% (CI − 0.1–0.2%)). It is unclear, however, why there are so few human cases of antibiotic resistance attributed to source drinking water, when compared to the other sources. In the measures of temporal association, the lowest amount of statistically significant associations was found between humans and source drinking water, but the difference between the same sources was not of the same scale as that seen in case attribution calculations. The overall results, however, do suggest that the relationship between human antibiotic resistance and their drinking water, in this setting, is more closely related to how humans interact with their drinking water than the source of the water itself. This is also the first study, to our knowledge, that has applied an attribution modelling to cases of human antibiotic resistance from environmental sources. Therefore, more research is required to further understand and clarify the contribution of each of these sources to human antibiotic resistance. However, this work does provide a promising start to use of mathematical modelling to understand and quantify the effects of environmental sources on human antibiotic resistance.

The level of temporal association, determined by Newey–West regression models, between the children and their environment, and the level of case attribution, estimated by *SourceR,* of each source to antibiotic resistance in children, support the concept of ‘one-health’. They highlight the overlapping nature of levels of antibiotic resistance in humans and that of the animal and environmental biospheres. The ‘one-health’ concept should therefore be used to inform interventions and policies to combat antibiotic resistance, as is already suggested by international organisations^1,2,33–35^. Several previous studies have shown evidence of transmission between these distinct disciplines^23,36–42^ and the theories are widely accepted and integrated into international plans for managing antibiotic resistance^1,7,9,43^. However, to date, this is the first study that has directly investigated the relationship between the three disciplines over time. Furthermore, we were able to analyse this relationship, with real-life observations, over real-time. To further understand these associations, more research that incorporates the observation over time is needed, as we showed a level of fluctuation between each time point. When observing the trend for ampicillin (see Fig. 3), there was an observable spike in resistance to all sources at time point 6 (Summer 2016) that we cannot explain. This may have occurred for many reasons, including, a change in antibiotic prescription, use or disposal in the area, or this may have been caused by an event or multiple events, such as the Simhastha Kumbh Mela; a religious pilgrimage and festival in the nearby city of Ujjain that occurred a few months prior to this collection point, but it is unclear what, if any, relationship that may have had.

When considering the temporal trends in antibiotic resistance, our results were mixed. We found, perhaps surprisingly, that where there was a statistically significant trend, they were more likely to be negative, suggesting a possible overall downward trend within the community. However, most of our results did not show a clear trend over time; 23 out of 87 regression coefficients were found to be statistically significant and relatively evenly spread out within the sources. It is also interesting that 18 of the 23 significant results came from just 7 antibiotics (including ESBL) and that within these individual antibiotics the direction of the trend was always only positive or negative. This suggests there has been a possible overall decrease in resistance in nalidixic acid, ciprofloxacin, tetracycline, sulfamethoxazole and ESBL, but an increase in resistance in ampicillin and amikacin. Our findings are similar of that found in another study from France, which found that most of the trends in antibiotic resistance in humans, pets and food-animals were either stationary or negative^30^. However, the consensus of the literature and guidance from international organisations is that antibiotic resistance is increasing globally^1,3,4,24,44^. More specifically, most publications suggest antibiotic resistance is increasing in India^25,45^. Our results, therefore, cannot be used in isolation to determine antibiotic resistance trends across the country, region or globally, but add to the existing evidence base. Fortunately, in the future, the quality of data, regarding over time trends of resistance, is likely to be improved by the implementation of the Global Antimicrobial Resistance Surveillance System from the World Health Organization enabling continuous monitoring^26,46^, although surveillance across all aspects of one-health and community data is required. Another point of consideration when comparing to other studies was that our study area was a rural community, with children as the study population. This may account for some of the differences in results, as most research is from tertiary centres and in adults^47–50^; for example, our participants were clinically well and assumed to be antibiotic naive, whereas adults and hospital patients are more likely to be or have been exposed to antibiotics. Although paediatric antibiotic resistance is itself of importance^47^, it is unclear if these results will remain similar for different age groups.

It is already established that antibiotic resistance increases with antibiotic use^1,4^, so an understanding of prescribing patterns is required to control the spread of antibiotic resistance^6^. The strength of selection for resistance differs depending on the balance of the cost and benefits of resistance^28^. It seems that antibiotic resistance confers a cost to the bacterium, which is expressed as diminished competitive ability when antibiotics are absent^51^. Therefore, where certain antibiotics have a reduced entry into the transmission pathways, a decline in resistance specific to that antibiotic may be seen^28,52^. Therefore, to understand the results of resistance patterns and trends better, they need to be studied alongside the local antibiotic prescription behaviours^6^. This is important in India, where antibiotic prescription practices vary across the country and require greater stewardship^53^. This is not within our remit, but briefly, the local rates of antibiotic prescription are high, across several hospital departments and while there is variation of type of antibiotic prescribed, they tend to be broad-spectrum and often not indicated^54–57^. Additionally, informal healthcare providers also prescribe high levels of antibiotics in the area^58^. Further, there is high antibiotic use in agriculture in India, although the exact figures for usage is unknown^59^. However, antibiotic consumption alone does not explain variations in antibiotic resistance, so other factors need considering from both a microbiological and from a one-health behavioural aspect, such as interaction of determinants of resistance, bystander selection^26,60^ or disposal of antibiotics. In keeping with ‘one health’, it is also important to consider agricultural practices, with regards to both antibiotic use, animal waste and subsequent wastewater flow^61–63^, with these factors highlighted with regards to antibiotic resistance in India^59^. These factors further underline the importance of multi-disciplinary and -sectorial work with close collaboration as part of a concerted effort^59^.

One of the strengths of this study is the collection of data over an extended period. It incorporates time into the analysis and recognises that the level of resistance at one point is dependent on previous findings. Temporal analysis, such as time series, has been used in previous studies into antibiotic resistance, but they have largely focused on comparing resistance to antibiotic use or the effects of antibiotic stewardship programmes^64–68^. However, none to date have analysed the association of resistance between different sources, incorporating temporality. The data was collected simultaneously and collated homogenously, which allowed the study of associations between the sources. Previous studies of antibiotic resistance that have included all three components of the ‘one health’ concept have been limited by not collecting data in this way, hence these studies only describe patterns and trends^30,31^. However, there are weaknesses within this study design as well. Whilst we believe this study offers a novel approach to attribution of human antibiotic resistance from environmental sources, this could have been strengthened further by incorporating genotypes into the model, possibly allowing a more precise measure of transmission, however this was not possible within this study. This is, in part, a reflection of the resources available, with the setting being a lower middle income country, and within this setting the utilisation of this tool may be more likely to be repeated with less expensive phenotypic analysis rather than genotypic. Further, this study phenotypically determined antibiotic resistance, as in clinical practice the greater interest is in the antibiotics and the groups of antibiotics to which there are resistance, rather than the mechanism of resistance. This is the first use of *SourceR* to calculate attribution for antibiotic resistance and using phenotypic measures of resistance rather than genotypic. It therefore may be prudent to regard the output as a suggestion of relative association between sources and humans of case attribution levels rather than an absolute calculation, this is highlighted by the variability within the results. Further, *SourceR* is designed for use with cross-sectional data, from which causality and directionality cannot be reliably determined. The number of samples collected at each time varied depending on the source and number of samples collected where *E. coli* was isolated. In total, 41 children were not available for all seven time points and samples were not received for a total of 63 times, but this represents only a small amount of the planned total samples for children (n = 875), with only 7.2% of samples not received. While power calculations for linear regressions and ANOVAs cannot be used in time series data^69^, based on simple statistics, the proportion of resistance is likely to be less accurate where smaller numbers of *E. coli* were isolated. However, Newey–West regression modelling of temporal data uses ordinary least squares^70^ which is robust and works well for small sample sizes^71^. A high number of statistical tests were performed; thus, we cannot rule out that some of the results are due to chance. However, the number of statistically significant results from the Newey–West calculations were higher than that expected by chance alone with a statistically significant *p*-value set at 0.05 (26.1% of calculations of association were significant and 26.4% of trends). No correction of multiple statistical tests has been done, as the analysis done here was multiple use of one simple test and it is the individual tests and their results are of importance^72^. The Kirby-Bauer disc diffusion method is robust for antibiotic susceptibility testing for all included antibiotics, other than colistin^27^. This was accounted for by simultaneous detection of the *mcr-1* resistance gene on all isolates^11,27^. Colistin was also included late in the study, with collection at three time points only and with a smaller number of samples. However, the aim of this inclusion was to get a baseline for colistin resistance in this community and as such limited analysis of over time trends and associations have been done and it was excluded from the attribution modelling.

In conclusion, there was an observable temporal association between human antibiotic resistance prevalence and that of the animals and environmental sources in proximity. The closest of these relationships appears to be between human antibiotic resistance levels and household drinking water. This is supported by case attribution modelling, with the highest levels of attribution to human cases of antibiotic resistance seen in household drinking water and animals. Our results add valuable data to the overall picture of over time trends, but perhaps more importantly we provide a platform to study the relationships between the resistance levels in the three pillars of the ‘one-health’ concept. Where there was a statistically significant over time trend in resistance pattern, most of them showed a negative trend. The exceptions were in ampicillin and amikacin, which showed positive trends in more than one of the sources studied.

## Methods

### Study design

This prospective cohort study collected data from different sources: child stool, animal (cattle, hen, dog, goat and horse) stool, household drinking water, source drinking water and wastewater sources. Both the human and animal stool samples were representative of commensal, enteric *E. coli*, due to clinical absence of infection. Samples were collected at regular, four-month intervals, for two years, totalling 7 time points.

### Study setting

The data was collected in the rural Palwa demographic surveillance site of Ruxmaniben Deepchand Gardi Medical College (RDGMC) Ujjain District, Madhya Pradesh, India. Madhya Pradesh, centrally located in India, is the second largest state and approximately 75% of the inhabitants live in rural areas^27^. As many as 80% of the healthcare workers, both formal and informal, are concentrated in one village within the surveillance site^6^, hereafter referred to as the central village.

### Sampling selection

Further details of sampling are outlined in previous studies^6,11,27^. In brief, the six villages that met the inclusion criteria were purposively selected from 60 villages. The inclusion criteria were: to be within 5 km of the central village; total village population of 500 people; more than 15 children aged between 1 and 3 years old available in the village and a transport time of less than 45 min to the RDGMC Central Research Laboratory from each village. Further inclusion criteria for the human participants were: a child aged between 1 and 3 years old; living in the village for at least the past year; planning on living in the village for the three upcoming years and willing to participate in the study. Subsequently, simple random sampling was performed to select a total sample of 125 children.

### Data collection

Details of the collection, transport and laboratory methods are outlined in previous studies^6,11,27^. The data was collected between August 2014 and September 2016. Sample collection was organised by trained research assistants. The data was collected in the three seasons experienced in the study setting; namely summer (March to June), monsoon (late June to September) and post-monsoon/winter (October to February). Each selected child provided a stool sample and a sample from their household’s drinking water storage vessel, which was collected by their parents or guardians. In each village, a sample of drinking water was collected from two source drinking water sites and a wastewater sample was collected from two discharge sites. The source drinking water sites are communal drinking water collection points in the village that are typically used to fill the household drinking water storage vessel. Stool samples from five animals (cattle, hen, dog, goat and horse) were collected in each village. The animals do not live within the households, but all commonly share the environment with the children.

Details of the preparation of the samples are also outlined in prior studies^6,73^. A total of six *E. coli* colonies were isolated from each sample. Antibiotic susceptibility testing was performed using the Kirby-Bauer disc diffusion method^74^, as per the current Clinical and Laboratory Standards Institute (CLSI) guidelines^75^. Antibiotics were chosen following the antibiotics used in the study area for gram-negative coliform infections and the CLSI guidelines^75^. The following antibiotics were chosen: ampicillin, cefotaxime, ceftazidime, cefepime, nalidixic acid, ciprofloxacin, nitrofurantoin, gentamicin, amikacin, tetracycline, tigecycline, imipenem, meropenem, co-trimoxazole, sulfamethoxazole and colistin. Measurements of colistin resistance were done by detecting *mcr-1* gene and by disc diffusion. These measurements were taken for the three last time points as colistin is not regularly used in this setting but was later included in order to have a baseline measurement of community resistance in this important, last-line antibiotic. Measurements of mono-resistance (resistance to one antibiotic) and multi-drug resistance (MDR) (resistance involving three or more antibiotics of three different groups) were undertaken. The intermediate isolates were then grouped together with resistant isolates and classified as resistant.

If full collection of data was not possible, for example if the participant left the study area, the household was excluded from the study. However, if one data collection sample was not received from the caregiver on the day of collection, they were marked as ‘Sample Not Received’ for that collection round but continued in the study. If the sample was not received or it was not possible to isolate *E. coli* in one of the samples then they were excluded from the analysis of the results of that time point.

### Statistical analysis

The first analysis of the trends and relationships was done using Stata Version 16.1 (StataCorp. College Station, TX, USA) and Excel (Microsoft Corp., Redmond, WA, USA). Firstly, the proportions and percentages of resistant isolates, per data collection point, per source, to each antibiotic were calculated. A measurement of the mean proportion of resistant isolates was made for each source, for each antibiotic. The data was grouped ordinally, by sample collection date, to create a data series of the percentage of resistance in each antibiotic at each of the seven times samples were collected. Newey–West regression modelling of temporal data^70^ was used to test the temporal trend of percentage of resistance for each antibiotic, for each source, against time. Newey–West can be used to estimate the covariance matrix of parameters of a regression where typical assumptions of a regression-model are not true. In this instance, it was used because the level of resistance at a time point is dependent on the previous time point. By using this method we incorporate time into the analysis. The command “tsset” was used in Stata to identify the collection point as an ordinal time variable for each Newey–West regression model. The dependent variable was the percentage of resistance at each time point for the antibiotic and source being calculated, with a separate model calculated for each antibiotic and source, and the independent variable was the collection point. A lag of one time point was used for each model. A total of 87 models were used to calculate the overtime trends of antibiotic resistance. The same method was used to assess the temporal association between proportion of resistance in human samples and the other sources individually. The collection point was used as the time variable in this model and the lag was one time point for each model. The dependent variable was the percentage of human antibiotic resistance to the antibiotic being calculated at each time point. The independent variable was of the percentage of resistance to the specified antibiotic at each time point for either the animal, household drinking water, source drinking water or wastewater sources. A separate model was created for each of these independent variables for each antibiotic. Thus, a total of 69 individual models were used to estimate the association of human antibiotic resistance to that of the other sources. Fitting of the attribution model was done via RStudio version. 4.0.2, using *sourceR*, which is available from the Comprehensive R Archive Network and released under a GPL-3 licence. The antibiotics were grouped as per their class to represent phenotypic resistance, this was defined as “Type” in the existing model and similarly “Cases” were defined as human antibiotic resistance^29^. Indicative priors were calculated with a Dirichlet process where the concentration parameter was one. A total of 1000 iterations were used for the MCMC chain, with a burn-in of 10,000 and thinning of 500, as used previously in *SourceR* modelling^29^. The measurements of human cases attributable to each environmental source and the proportion of cases attributable to each source were extracted from the model for each time point and location and expressed as medians. Means and 95% confidence intervals of these results were calculated as well as percentages of attribution, giving an overall figure of human cases attributable to each source. Colistin was excluded from the modelling, as due to incomplete collection it was not compatible with the rest of the model. Measures of multi-drug resistance were also excluded from the modelling, as it is not a measure of resistance to a specific antibiotic or group of antibiotics.

### Ethical considerations.

Ethical review number: 2013/07/17-311 (Institutional Ethics committee RDGMC, Ujjain, India). Oral and written informed consent was obtained for each participant from a parent or legal guardian. There were no harmful or invasive procedures performed in this study. All children identified as needing medical care were referred to and treated by paediatric services in collaboration with the RDGMC.

## Supplementary Information

Supplementary Information.

## Data Availability

The datasets generated during and/or analysed during the current study are available from the corresponding author on reasonable request.
