# Supplementary material for: Trends, relationships and case attribution of antibiotic resistance between children and environmental sources in rural India
Source: Sci Rep. 2021 Nov 19;11:22599. doi: 10.1038/s41598-021-01174-w (PMC8604955; doi:10.1038/s41598-021-01174-w)
Supplement: Supplementary file 1 — Supplementary Information. [file 41598_2021_1174_MOESM1_ESM.docx]

**Supplementary Table 1**

Table 1.A The number of resistant E.coli isolates to each antibiotic, per the total of E.coli isolated, also presented as a percentage, from human samples, at each time point.

| *ABX* | Monsoon 2014 | Winter 2015 | Summer 2015 | Monsoon 2015 | Winter 2016 | Summer 2016 | Monsoon 2016 |
| --- | --- | --- | --- | --- | --- | --- | --- |
| AMP | 379/632  60.0% | 365/655  55.7% | 448/675  66.4% | 377/704  53.6% | 339/676  50.1% | 671/699  96.0% | 611/693  88.2% |
| CTX | 313/632  49.5% | 253/655  38.6% | 322/675  47.7% | 318/704  45.2% | 263/676  38.9% | 260/699  37.2% | 333/693  48.1% |
| CAZ | 277/632  43.8% | 219/655  33.4% | 280/675  41.5% | 217/704  30.8% | 259/676  38.3% | 256/699  36.6% | 326/693  47.0% |
| CPM | 224/632  35.4% | 157/655  24.0% | 172/675  25.5% | 134/704  19.0% | 185/676  27.4% | 230/699  32.9% | 350/693  50.5% |
| NA | 357/632  56.5% | 352/655  53.7% | 327/675  48.4% | 366/704  52.0% | 342/676  50.6% | 324/699  46.4% | 391/693  56.4% |
| CIP | 135/632  21.4% | 103/655  15.7% | 93/675  13.8% | 123/704  17.5% | 119/676  17.6% | 85/699  12.2% | 181/693  26.1% |
| NIT | 42/632  6.6% | 26/655  4.0% | 22/675  3.3% | 40/704  5.7% | 14/676  2.1% | 19/699  2.7% | 30/693  4.3% |
| GEN | 19/632  3.0% | 19/655  2.9% | 19/675  2.8% | 20/704  2.8% | 30/676  4.4% | 11/699  1.6% | 9/693  1.3% |
| AK | 11/632  1.7% | 6/655  0.9% | 22/675  3.3% | 27/704  3.8% | 11/676  1.6% | 39/699  5.6% | 22/693  3.2% |
| TE | 148/632  23.4% | 146/655  22.3% | 123/675  18.2% | 189/704  26.8% | 163/676  24.1% | 90/699  12.9% | 181/693  26.1% |
| TGC | 1/632  0.2% | 3/655  0.5% | 0/675  0.0% | 3/704  0.4% | 0/676  0.0% | 3/699  0.4% | 0/693  0.0% |
| IPM | 8/632  1.3% | 12/655  1.8% | 137/675  20.3% | 145/704  20.6% | 126/676  18.6% | 19/699  2.7% | 5/693  0.7% |
| MRP | 70/632  11.1% | 73/655  11.1% | 37/675  5.5% | 52/704  7.4% | 54/676  8.0% | 69/699  9.9% | 156/693  22.5% |
| COT | 163/632  25.8% | 134/655  20.5% | 205/675  30.4% | 205/704  29.1% | 176/676  26.0% | 169/699  24.2% | 240/693  34.6% |
| SM | 179/632  28.3% | 128/655  19.5% | 216/675  32.0% | 261/704  37.1% | 189/676  28.0% | 149/699  21.3% | 212/693  30.6% |
| CL | N/A | N/A | N/A | N/A | 1/379  0.3% | 2/699  0.3% | 2/507  0.4% |
| ESBL | 285/632  45.1% | 241/655  36.8% | 300/675  44.4% | 291/704  41.3% | 216/676  32.0% | 222/699  31.8% | 228/693  32.9% |
| MDR | 310/632  49.1% | 264/655  40.3% | 342/675  50.7% | 360/704  51.1% | 311/676  46.0% | 317/699  45.4% | 406/693  58.6% |

Supplementary Table 1.B The number of resistant E.coli isolates to each antibiotic, per the total of E.coli isolated, also presented as a percentage, from animal samples, at each time point.

| *ABX* | Monsoon 2014 | Winter 2015 | Summer 2015 | Monsoon 2015 | Winter 2016 | Summer 2016 | Monsoon 2016 |
| --- | --- | --- | --- | --- | --- | --- | --- |
| AMP | 40/151  26.5% | 39/167  23.4% | 32/159  20.1% | 33/168  19.6% | 37/155  23.9% | 151/162  93.2% | 108/168  64.3% |
| CTX | 23/151  15.2% | 15/167  9.0% | 26/159  16.4% | 25/168  14.9% | 35/155  22.6% | 20/162  12.3% | 21/168  12.5% |
| CAZ | 20/151  13.2% | 15/167  9.0% | 25/159  15.7% | 25/168  14.9% | 33/155  21.3% | 31/162  19.1% | 21/168  12.5% |
| CPM | 13/151  8.6% | 14/167  8.4% | 13/159  8.2% | 11/168  6.5% | 16/155  10.3% | 23/162  14.2% | 20/168  11.9% |
| NA | 19/151  12.6% | 26/167  15.6% | 34/159  21.4% | 31/168  18.5% | 37/155  23.9% | 30/162  18.5% | 26/168  15.5% |
| CIP | 6/151  4.0% | 11/167  6.6% | 16/159  10.1% | 12/168  7.1% | 9/155  5.8% | 6/162  3.7% | 4/168  2.4% |
| NIT | 11/151  7.3% | 4/167  2.4% | 19/159  11.9% | 8/168  4.8% | 1/155  0.6% | 0/162  0.0% | 7/168  4.2% |
| GEN | 1/151  0.7% | 3/167  1.8% | 0/159  0.0% | 3/168  1.8% | 0/155  0.0% | 0/162  0.0% | 0/168  0.0% |
| AK | 1/151  0.7% | 2/167  1.2% | 3/159  1.9% | 1/168  0.6% | 3/155  1.9% | 6/162  3.7% | 8/168  4.8% |
| TE | 9/151  6.0% | 16/167  9.6% | 23/159  14.5% | 11/168  6.5% | 4/155  2.6% | 8/162  4.9% | 13/168  7.7% |
| TGC | 2/151  1.3% | 1/167  0.6% | 0/159  0.0% | 0/168  0.0% | 0/155  0.0% | 1/162  0.6% | 3/168  1.8% |
| IPM | 0/151  0.0% | 4/167  2.4% | 52/159  32.7% | 19/168  11.3% | 17/155  11.0% | 1/162  0.6% | 1/168  0.6% |
| MRP | 8/151  5.3% | 7/167  4.2% | 14/159  8.8% | 8/168  4.8% | 10/155  6.5% | 7/162  4.3% | 5/168  3.0% |
| COT | 5/151  3.3% | 16/167  9.6% | 4/159  2.5% | 16/168  9.5% | 7/155  4.5% | 10/162  6.2% | 6/168  3.6% |
| SM | 31/151  20.5% | 48/167  28.7% | 21/159  13.2% | 23/168  13.7% | 25/155  16.1% | 17/162  10.5% | 7/168  4.2% |
| CL | N/A | N/A | N/A | N/A | 0/98  0.0% | 0/162  0.0% | 0/114  0.0% |
| ESBL | 17/151  11.3% | 12/167  7.2% | 20/159  12.6% | 25/168  14.9% | 26/155  16.8% | 26/162  16.0% | 21/168  12.5% |
| MDR | 17/151  11.3% | 22/167  13.2% | 35/159  22.0% | 24/168  14.3% | 35/155  22.6% | 27/162  16.7% | 16/168  9.5% |

Supplementary Table 1.C The number of resistant E.coli isolates to each antibiotic, per the total of E.coli isolated, also presented as a percentage, from household drinking water samples, at each time point.

| *ABX* | Monsoon 2014 | Winter 2015 | Summer 2015 | Monsoon 2015 | Winter 2016 | Summer 2016 | Monsoon 2016 |
| --- | --- | --- | --- | --- | --- | --- | --- |
| AMP | 201/627  32.1% | 158/529  29.9% | 202/554  36.5% | 177/674  26.3% | 118/425  27.8% | 492/552  89.1% | 363/456  79.6% |
| CTX | 145/627  23.1% | 100/529  18.9% | 104/554  18.85 | 130/674  19.3% | 79/425  18.6% | 71/552  12.9% | 86/456  18.9% |
| CAZ | 117/627  18.7% | 90/529  17.0% | 99/554  17.9% | 112/674  16.6% | 85/425  20.0% | 94/552  17.0% | 81/456  17.8% |
| CPM | 88/627  14.0% | 71/529  13.4% | 68/554  12.3% | 68/674  10.1% | 35/425  8.2% | 48/552  8.7% | 104/456  22.8% |
| NA | 109/627  17.4% | 98/529  18.5% | 76/554  13.7% | 146/674  21.7% | 54/425  12.7% | 56/552  10.1% | 74/456  16.2% |
| CIP | 66/627  10.5% | 41/529  7.8% | 22/554  4.0% | 43/674  6.4% | 12/425  2.8% | 19/552  3.4% | 22/456  4.8% |
| NIT | 48/627  7.7% | 27/529  5.1% | 12/554  2.2% | 30/674  4.5% | 11/425  2.6% | 12/552  2.2% | 47/456  10.3% |
| GEN | 22/627  3.5% | 14/529  2.6% | 7/554  1.3% | 26/674  3.9% | 1/425  0.2% | 1/552  0.2% | 3/456  0.7% |
| AK | 15/627  2.4% | 10/529  1.9% | 27/554  4.9% | 24/674  3.6% | 5/425  1.2% | 10/552  1.8% | 9/456  2.0% |
| TE | 92/627  14.7% | 53/529  10.0% | 55/554  9.9% | 93/674  13.8% | 36/425  8.5% | 25/552  4.5% | 39/456  8.6% |
| TGC | 6/627  1.0% | 2/527  0.4% | 2/554  0.4% | 2/674  0.3% | 1/425  0.2% | 1/552  0.2% | 2/456  0.4% |
| IPM | 9/627  1.4% | 11/529  2.1% | 100/554  18.1% | 132/674  19.6% | 67/425  15.8% | 9/552  1.6% | 5/456  1.1% |
| MRP | 56/627  8.9% | 26/529  4.9% | 11/554  2.0% | 29/674  4.3% | 7/425  1.6% | 18/552  3.3% | 30/456  6.6% |
| COT | 61/627  9.7% | 30/529  5.7% | 53/554  9.6% | 84/674  12.5% | 51/425  12.0% | 27/552  4.9% | 48/456  10.5% |
| SM | 111/627  17.7% | 67/529  12.7% | 102/554  18.4% | 156/674  23.1% | 72/425  16.9% | 65/552  11.8% | 54/456  11.8% |
| CL | N/A | N/A | N/A | N/A | 3/243  1.2% | 0/546  0.0% | 1/336  0.3% |
| ESBL | 112/627  17.9% | 88/529  16.6% | 90/554  16.2% | 120/674  17.8% | 61/425  14.4% | 70/552  12.7% | 54/456  11.8% |
| MDR | 141/627  22.5% | 95/529  18.0% | 112/554  20.2% | 143/674  21.2% | 76/425  17.9% | 76/552  13.8% | 99/456  21.7% |

Supplementary Table 1.D The number of resistant E.coli isolates to each antibiotic, per the total of E.coli isolated, also presented as a percentage, from source drinking water samples, at each time point.

| *ABX* | Monsoon 2014 | Winter 2015 | Summer 2015 | Monsoon 2015 | Winter 2016 | Summer 2016 | Monsoon 2016 |
| --- | --- | --- | --- | --- | --- | --- | --- |
| AMP | 15/34  44.1% | 11/22  50.0% | 15/34  44.1% | 16/62  25.8% | 7/26  26.9% | 49/54  90.7% | 17/30  56.7% |
| CTX | 12/34  35.4% | 6/22  27.3% | 2/34  5.9% | 8/62  12.9% | 5/26  19.2% | 7/54  13.0% | 2/30  6.7% |
| CAZ | 12/34  35.3% | 6/22  27.3% | 2/34  5.9% | 8/62  12.9% | 4/26  15.4% | 9/54  16.7% | 2/30  6.7% |
| CPM | 10/34  29.4% | 6/22  27.3% | 2/34  5.9% | 5/62  8.1% | 2/26  7.7% | 6/54  11.1% | 9/30  30.0% |
| NA | 14/34  41.2% | 10/22  45.5% | 9/34  26.5% | 16/62  25.8% | 4/26  15.4% | 10/54  18.5% | 2/30  6.7% |
| CIP | 6/34  17.6% | 3/22  13.6% | 4/34  11.8% | 6/62  9.7% | 2/26  7.7% | 2/54  3.7% | 1/30  3.3% |
| NIT | 2/34  5.9% | 4/22  18.2% | 2/34  5.9% | 3/62  4.8% | 3/26  11.5% | 1/54  1.9% | 3/30  10.0% |
| GEN | 0/34  0.0% | 0/22  0.0% | 1/34  2.9% | 6/62  9.7% | 0/26  0.0% | 0/54  0.0% | 0/30  0.0% |
| AK | 1/34  2.9% | 0/22  0.0% | 6/34  17.6% | 2/62  3.2% | 0/26  0.0% | 0/54  0.0% | 0/30  0.0% |
| TE | 13/34  38.2% | 9/22  40.9% | 4/34  11.8% | 10/62  16.1% | 4/26  15.4% | 4/54  7.4% | 0/30  0.0% |
| TGC | 3/34  8.8% | 1/22  4.5% | 0/34  0.0% | 0/62  0.0% | 0/26  0.0% | 0/54  0.0% | 0/30  0.0% |
| IPM | 1/34  2.9% | 0/22  0.0% | 11/34  32.4% | 14/62  22.6% | 3/62  4.8% | 0/54  0.0% | 0/30  0.0% |
| MRP | 7/34  20.6% | 8/22  36.4% | 1/34  2.9% | 1/62  1.6% | 2/26  7.7% | 1/54  1.9% | 1/30  3.3% |
| COT | 5/34  14.7% | 3/22  13.6% | 1/34  2.9% | 9/62  14.5% | 3/26  11.5% | 3/54  5.6% | 3/30  10.0% |
| SM | 9/34  26.5% | 10/22  45.5% | 4/34  11.8% | 10/62  16.1% | 12/26  46.2% | 8/54  14.8% | 3/30  10.0% |
| CL | N/A | N/A | N/A | N/A | 0/13  0.0% | 0/48  0.0% | 0/28  0.0% |
| ESBL | 11/34  32.4% | 6/22  27.3% | 2/34  5.9% | 8/62  12.9% | 4/26  19.2% | 8/54  14.8% | 0/30  0.0% |
| MDR | 13/34  38.2% | 10/22  45.5% | 8/34  23.5% | 12/62  19.4% | 5/26  19.2% | 7/54  13.0% | 3/30  10.0% |

Supplementary Table 1.E The number of resistant E.coli isolates to each antibiotic, per the total of E.coli isolated, also presented as a percentage, from wastewater samples, at each time point.

| *ABX* | Monsoon 2014 | Winter 2015 | Summer 2015 | Monsoon 2015 | Winter 2016 | Summer 2016 | Monsoon 2016 |
| --- | --- | --- | --- | --- | --- | --- | --- |
| AMP | 31/74  41.9% | 32/68  47.1% | 33/67  49.3% | 20/72  27.8% | 14/71  19.7% | 69/71  97.3% | 38/65  58.5% |
| CTX | 23/74  31.1% | 23/68  33.8% | 2/67  3.0% | 14/72  19.4% | 15/71  21.1% | 31/71  43.7% | 14/65  21.5% |
| CAZ | 18/74  24.3% | 23/68  33.8% | 2/67  3.0% | 13/72  18.1% | 14/71  19.7% | 34/71  47.9% | 12/65  18.5% |
| CPM | 14/74  18.9% | 16/68  23.5% | 1/67  1.5% | 12/72  16.7% | 9/71  12.7% | 23/71  32.4% | 22/65  33.8% |
| NA | 32/74  43.2% | 35/68  51.5% | 16/67  23.9% | 32/72  44.4% | 19/71  26.8% | 25/71  35.2% | 12/65  18.5% |
| CIP | 12/74  16.2% | 19/68  27.9% | 3/67  4.5% | 7/72  9.7% | 8/71  11.3% | 8/71  11.3% | 3/65  4.6% |
| NIT | 1/74  1.4% | 2/68  2.9% | 1/67  1.5% | 6/72  8.3% | 3/71  4.2% | 4/71  5.6% | 2/65  3.1% |
| GEN | 2/72  2.8% | 3/68  4.4% | 0/67  0.0% | 2/72  2.8% | 2/71  2.8% | 1/71  1.4% | 0/65  0.0% |
| AK | 1/74  1.4% | 0/68  0.0% | 3/67  4.5% | 1/72  1.4% | 3/71  4.2% | 1/71  1.4% | 2/65  3.1% |
| TE | 15/74  20.3% | 14/68  20.6% | 2/67  3.0% | 9/72  12.5% | 11/71  15.5% | 5/71  7.0% | 8/65  12.3% |
| TGC | 1/74  1.4% | 2/68  2.9% | 0/67  0.0% | 0/72  0.0% | 0/71  0.0% | 0/71  0.0% | 0/65  0.0% |
| IPM | 5/74  6.8% | 3/68  4.4% | 7/67  10.4% | 12/72  16.7% | 19/71  26.8% | 0/71  0.0% | 0/65  0.0% |
| MRP | 9/74  12.2% | 17/68  25.0% | 0/67  0.0% | 5/72  6.9% | 7/71  9.9% | 4/71  5.6% | 6/65  9.2% |
| COT | 11/74  14.9% | 9/68  13.2% | 5/67  7.5% | 11/72  15.3% | 13/71  18.3% | 11/71  15.5% | 2/65  3.1% |
| SM | 21/74  28.4% | 12/68  17.6% | 8/67  11.9% | 11/72  15.3% | 14/71  19.7% | 10/71  14.1% | 3/65  4.6% |
| CL | N/A | N/A | N/A | N/A | 0/45  0.0% | 0/71  0.0% | 0/41  0.0% |
| ESBL | 18/74  24.3% | 19/68  27.9% | 2/67  3.0% | 12/72  16.7% | 7/71  9.9% | 27/71  38.0% | 6/65  9.2% |
| MDR | 26/74  35.1% | 22/68  32.4% | 7/67  10.4% | 19/72  26.4% | 21/71  29.6% | 27/71  38.0% | 12/65  18.5% |

*Abbreviations:* AMP = AMPICILLIN; CTX = CEFOTAXIME; CAZ = CEFTAZIDIME; CPM = CEFEPIME; NA = NALIDIXIC ACID; CIP = CIPROFLOXACIN; NIT = NITROFURANTOIN; GEN = GENTAMICIN; AK = AMIKACIN; TE = TETRACYCLINE; TGC = TIGECYCLINE; IPM = IMIPENEM; MRP = MEROPENEM; COT = CO-TRIMOXAZOLE; SM = SULPHAMETHIAZOLE; CL = COLISTIN; ESBL = EXTENDED SPECTRUM BETA LACTAMASE; MDR = MULTI DRUG RESISTANT

**Supplementary Figure 1**

*Abbreviations:* AMP = AMPICILLIN; CTX = CEFOTAXIME; CAZ = CEFTAZIDIME; CPM = CEFEPIME; NA = NALIDIXIC ACID; CIP = CIPROFLOXACIN; NIT = NITROFURANTOIN; GEN = GENTAMICIN; AK = AMIKACIN; TE = TETRACYCLINE; TGC = TIGECYCLINE; IPM = IMIPENEM; MRP = MEROPENEM; COT = CO-TRIMOXAZOLE; SM = SULPHAMETHIAZOLE; CL = COLISTIN; ESBL = EXTENDED SPECTRUM BETA LACTAMASE; MDR = MULTI DRUG RESISTANT

Supplementary Figure 1. Mean percentage of isolates resistant per source, per antibiotic.
